# Clinical management methods for out-patients with alcohol dependence

**DOI:** 10.1186/1747-597X-1-5

**Published:** 2006-02-01

**Authors:** Bertrand Nalpas, Florence Matelak, Sandrine Martin, Isabelle Boulze, Jean-Louis Balmes, Corinne Crouzet

**Affiliations:** 1 Inserm, U370, Faculté de Médecine Necker, 154 rue de Vaugirard, Paris, F-75015, France; 2 ANPAA30, 539B Avenue Jean Prouvé, Nîmes, F-30900, France; 3 Laboratoire Mémoire et cognition, EA 3021, Université Montpellier I, Montpellier, F-34000, France; 4 Service d'Addictologie, CHU CAREMEAU, Av du Pr R. Debré, Nîmes, F-30900, France

## Abstract

**Background:**

In France outpatient centres for the care of alcoholics are healthcare establishments providing medical, psychological and social support. Although they meet the practical needs of these patients, their degree of use in each of these domains and the respective mobilisation of different skills by the care team are not well understood. Our aim was therefore to determine in detail the management involved as a function of the severity of alcohol dependence. For this purpose, all the procedures involved were compiled in a thesaurus describing its type (psychological, medical, social, reception), its scheduled or unscheduled nature, its method (face-to-face, telephone, letter) and its duration. The severity of dependence was evaluated using the Addiction Severity Index (ASI).

**Results:**

45 patients were included and followed-up during 291 ± 114 days. The mean initial ASI scores (± SD) were: medical (M) = 0.39 ± 0.3, working-income (ER) = 0.5 ± 0.3, alcohol (A) = 0.51 ± 0.2, illicit drugs (D) = 0.07 ± 0.08, legal (L) = 0.06 ± 0.13, familial and social environment (FS) = 0.34 ± 0.26, psychological (P) = 0.39 ± 0.22. The total number of procedures was 1341 (29.8 per patient) corresponding to 754.4 hours (16.7 per patient). The intensity of management peaked during the first month of treatment, and then declined rapidly; the maximum incidence of abstinence was observed during the 3rd month of management. Interviews with patients, group therapy and staff meetings represented 68.7%, 9.9% and 13.9% of all procedures, respectively. In patients with severe dependence, as compared to moderate, management was twice as intense in the psychological and social domains, but not in the medical domain.

The ASI questionnaire was completed a second time by 24 patients, after an average of 3.2 months. The improvement was significant in the M, A, D and P domains only.

**Conclusion:**

This study provided an overview of the methods employed in managing a sample of patients consulting an alcoholism centre in line with standards for medical, psychological and social establishments. The predominance of the social and psychological domains over the medical domain was clearly established. Relapses were common after the third month of treatment, but a remobilisation of teams made it possible to contain them. These results provide a framework for discussions on the organisation of healthcare systems and highly suggest that staff need to maintain a constant level of care throughout the treatment process.

## Background

In France, care for alcoholic outpatients is provided by specialized centres which group together physicians trained in addiction problems, psychologists and social workers. These centres, the CCAA (Centre de Cure Ambulatoire en Alcoologie, or outpatient centres for the care of alcoholics), are funded by the National Social Security system and no payment is required from patients [1]. As far as we know, similar centres are implemented in other European countries such as Germany and Portugal.

Like all public healthcare structures, the CCAA are asked to produce an annual Activities Report, which is a crucial element in subsequent budget allocation. Activities are usually described in terms of the number of new and existing patients seen during the year and also the number of medical, psychological and social procedures carried out. These raw data are probably sufficient to ensure financial estimations but they cannot be used to accurately assess activities, as they take no account of the type of patients involved and the severity of addiction. Indeed, each patient reflects a different story of alcohol dependence and alcohol-related complications, so that some may require more medical care while others will need additional psychological and/or social support. Moreover, it is reasonable to hypothesize that more severe addiction will require a greater degree of care.

Although its activities are an important parameter for a given CCAA because they govern its financial resources, such a criteria provides no information as to the efficiency of treatment programmes. Insofar as the principal objective of any healthcare centre is to improve the health status of its patients, efficiency needs to be carefully analyzed in order to assess the relevance of the programmes implemented.

Although it is quite simple to determine activities in terms of the number of patients and procedures, that is not the case regarding efficiency. Indeed, it is now well accepted that stopping drinking is only one parameter for improvement among many others concerning a patient's medical, psychological and social status; the two latter factors are crucial as they are closely related to the occurrence of relapses [2]. In order to measure efficiency, therefore, a tool must take account of both the severity of addiction and its consequences and the need to adapt to changing circumstances. One of the most widely used tools in this regard is the Addiction Severity Index (ASI) [3], use of which has been validated in several countries. However, in France, the ASI is not applied at present in clinical practice as it considered to be too time-consuming. The aim of our study was therefore to describe the management of alcoholics in detail, with respect to healthcare procedures, the time required to complete them and their efficiency as determined by changes to ASI scores in a group of patients attending two CCAA located in the same geographical area.

## Results

### General characteristics of patients

The 45 patients included in the study comprised 30 men and 15 women with a mean age of 44.8 ± 10.5 years. Thirteen of them (28.9%) were unemployed. Their mean duration of alcohol abuse was 16.3 ± 10.4 years and their mean daily alcohol consumption was 175 ± 52 g. Familial alcoholism was present in more than half of the patients (55.5%) and 14 (31.1%) had been sentenced for driving whilst drunk. Most patients reported a recent or past history of psychological disorders: severe depression in 29 and anxiety in 38.

### ASI scores

**Figure 1:**
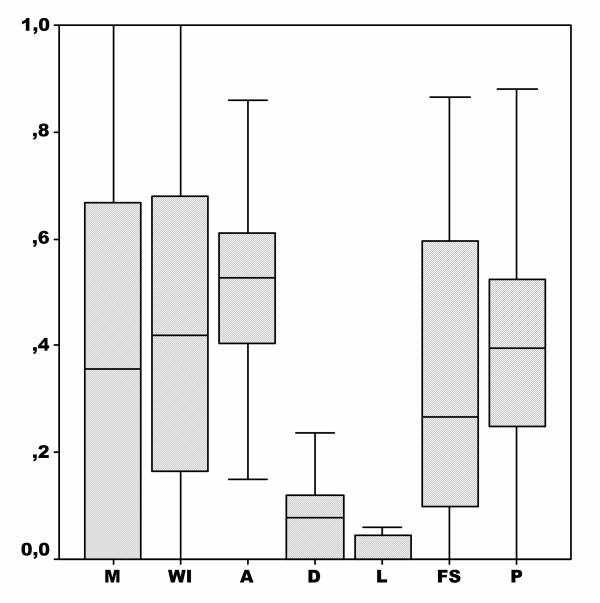
Boxplots of initial ASI sub-scores in the 45 studied patients.

The 45 patients included in the study completed the ASI questionnaire. The results of composite scores and the global mean scores are shown in Figure 1. All patients were alcoholics who had few problems with illicit drug use and no criminal record. The effects of their alcohol consumption were particularly marked in the psychological and social domains.

### Management

The mean duration of follow-up was 291 ± 114 days (median: 274 days); however, according to the study design, procedures carried out after the twelfth month of follow-up were right-censored. During the study period, 1341 procedures were carried out, giving a mean number per patient of 29.8 (median: 26); the corresponding number of hours was 754.38, i.e. 16.76 hours per patient (median: 9.9) (Table 1).

**Table 1:** Procedures and hours of care provided for the 45 patients during the study period

	Act	Hours
No. of patients	45	45
Sum	1341,00	754,39
Mean/patient	29,80	16,76
SD	21,53	18,86
Median	26,00	9,97
Minimum	2,00	1,00
Maximum	113,00	117,80
Percentiles		
25	14,00	6,62
50	26,00	9,97
75	36,50	22,91

**Figure 2:**
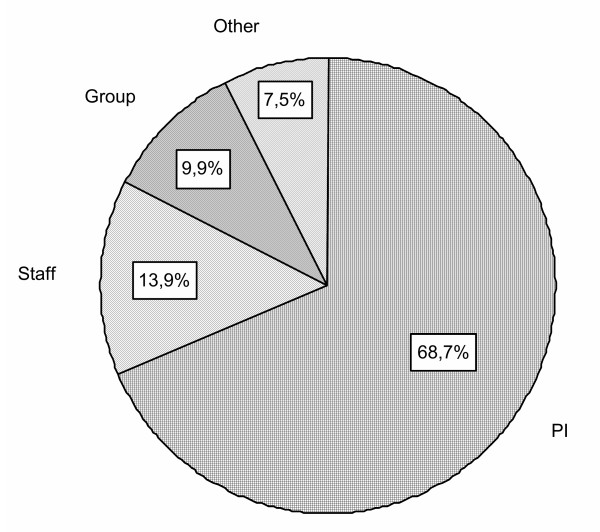
Distribution of types of intervention (PI = patient interview; Staff = staff meeting; Group = group therapy; Other = Family interview + administrative procedures + support + visits).

Most management consisted in interviews with patients (68.7% of all procedures) and then group therapy (9.9%) and administration (3.2%); team meetings accounted for 13.9% of procedures (Fig. 2).

**Figure 3:**
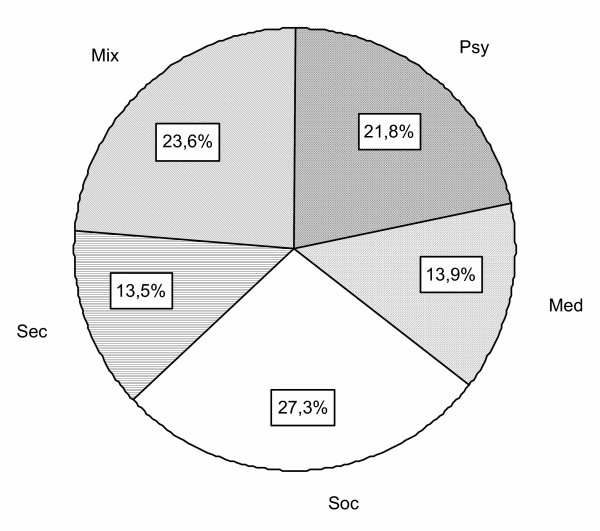
Distribution of procedures as a function of domain (Psy = psychological; Med = Medical; Soc = Social; Mix = several domains).

Analysis as a function of domain showed that procedures relative to the social domain were the most frequent (27.3% of procedures), followed by (in decreasing order) psychological procedures (21.8%), administrative (13.5%) and medical procedures (13.9%); those involving several domains (i.e. medical, psychological and social) corresponded to staff meetings and accounted for 23.6% of the total (Fig. 3).

82% of procedures were carried out face to face, 15.1% by telephone and 2.9% by letter.

Medical and psychological interviews were always scheduled; in contrast, half of those in the social domain were performed without a prior appointment. Almost all phone calls were unscheduled and originated from the patient (data not shown).

Under the classification described above, dependence was moderate in 22 patients and severe in 23. The duration of follow-up did not differ as a function of severity (8.8 months ± 2.9 versus 8.9 ± 2.6; Wilcoxon, W = 527, z = -0.046, p = 0.9). However, when dependence was severe, management was significantly more intense, whether this was expressed in terms of the number of procedures (37.1 ± 24.5 versus 22.2 ± 14.9; Wilcoxon, W = 409.5, z = -2.19, p = 0.02) or in the total management time per patient throughout the period (22.7 hours ± 23.0 versus 10.6 ± 9.1; Wilcoxon, W = 382, z = -2.81, p = 0.005).

Analysis according to the area of intervention showed that patients with severe dependence required more psychological support, with a mean of 8.7 ± 8.2 procedures during the study period, when compared to 4.1 ± 3.7 (Wilcoxon, W = 420.5, z = -1.95, p = 0.05) for those with moderate dependence; the same applied in the social domain (11.4 ± 12.5 versus 4.7 ± 4.9, Wilcoxon, W = 393, z = -2.57, p = 0.01) but not in the medical domain (4.7 ± 3.5 versus 3.5 ± 1.9, Wilcoxon, W = 452, z = -1.23, p = 0.21). The increased time spent arose from an increase in patient interviews (Wilcoxon, W= 391, z = -2.6, p = 0.009), family interviews (Wilcoxon, W = 425.5, z = -2.3, p = 0.02), administrative procedures (Wilcoxon, W = 393, z = -2.94, p = 0.003), and staff meetings (Wilcoxon, W = 398, z = -2.4, p = 0.01) while time devoted to group therapy, support and visits did not change significantly (data not shown).

**Figure 4:**
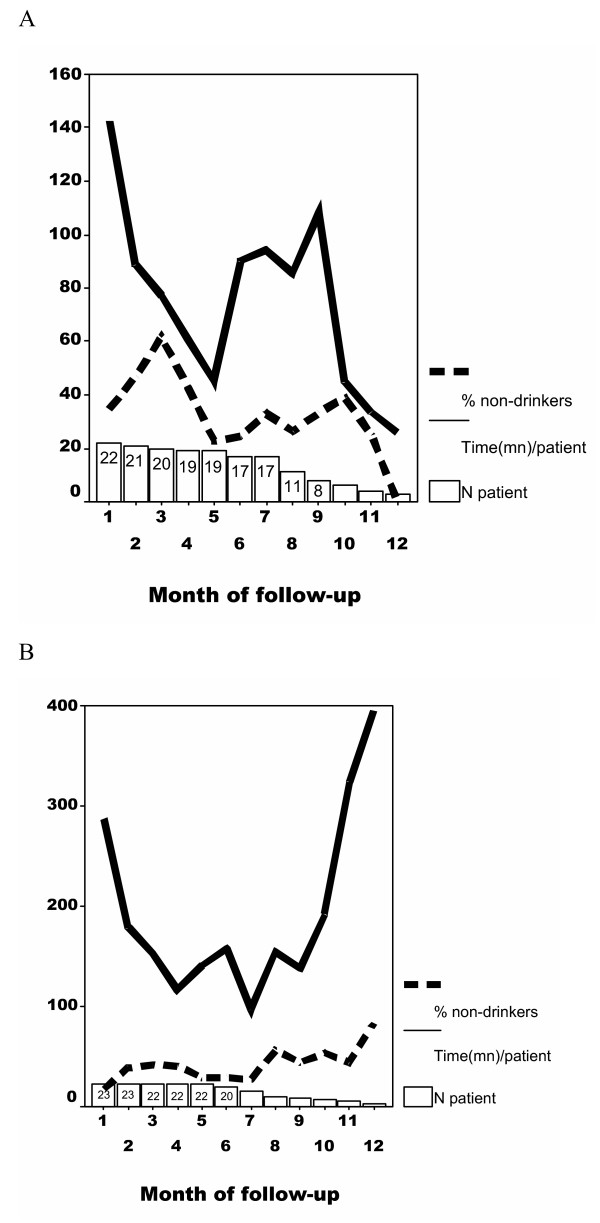
Intensity of care (mean intervention time/patient) as a function of the month of follow-up in patients with moderate (4A) or severe (4B) dependence.

The data collection method used during this study made it possible to achieve a detailed analysis of team involvement as a function of the month of treatment. The results obtained as a function of the severity of dependence are shown in Figures 4A and 4B. In both groups (moderate dependence and severe dependence) the intensity of management expressed as a mean time per patient reached its peak during the first month and then declined rapidly until around the 4th or 5th month; during this period, the proportion of abstinent patients increased regularly until the third month, and then declined again.

As from months 4–5, a marked increase was seen in the intensity of management, superimposed on an improvement in the proportion of abstinent patients, particularly amongst those whose dependence was moderate.

A large number of patients themselves discontinued management as from months 6–7, thus rendering uncertain any interpretation of the results; however, it could be seen in that in patients with severe dependence, this reduction was not accompanied by a reduction in care, because a marked increase was seen in the time devoted to those who continued to be followed.

Only twenty-four patients agreed to complete the ASI questionnaire a second time, after a mean interval of 3.2+/-0.5 months. The results could be interpreted for 21 patients. The differences in scores (value at second completion – value at first completion) are shown in Table 3. An improvement was defined by a negative difference, because according to the ASI, "normal" is equal to 0. Using Wilcoxon test for paired values a statistically significant improvement was observed with respect to the medical (z = -2.45, p = 0.01), alcohol (z = -2.91, p = 0.004), drugs (z = -2.2, p = 0.02) and psychological (z = -2.01, p = 0.04) domains. As for the social and familial environment, the difference was close to significance (z = -1.76, p = 0.08); however, no trends were observed with respect to work-income or legal status. Analysis after adjustment to the initial severity of dependence demonstrated improved results when the initial dependence was severe, but because of the small sample size, these changes were not significant.

## Discussion

This study enabled an in-depth analysis of the management of patients with alcohol problems, both in terms of the time devoted to each patient requesting help and the methods of management involved in the medical, psychological and social domains. Indeed, CCAAs aim to provide this multiple management, even though previously, the predominance of one domain over others and thus the mobilisation of resources in each domain was not fully understood.

The first observation was that this study enabled an a posteriori evaluation of the psychosocial model which characterises CCAA. Indeed, teams are involved in all three domains, although not to the same extent. The social domain accounts for the highest number of procedures and the most time, a result which should be interpreted from two angles: firstly, the social problems experienced by alcoholics consulting these centres are real and frequent, as witnessed by the scores for the work-income and social and family environment components of the ASI score; this fits well with the results of a recent survey conducted in the same geographical area showing that social workers considered that alcoholism was the most common problem encountered in the general population [4]. The social therapy of dependent patients has already been emphasised [5,6] but its actual role in overall management was not measured. Secondly, the offices in CCAA are usually manned by social workers, who are therefore in the front line if patients present themselves unexpectedly. This was fully verified when a comparison was made between scheduled interviews (defined as a direct contact with the subject) and unscheduled interviews, in the three areas of psychological, medical and social support. Although practically all interviews in the first two domains were always scheduled, half of those of a social work nature were unscheduled, meaning that patients availed themselves of the reception teams in an impromptu manner, thus confirming the usefulness of the opportunities they have to call in at any time.

Psychological support was the second most important component of management, taking up nearly 22% of time (not including team meetings). Although this aspect will vary as a function of the staff present in a centre, nonetheless the centres where this survey was carried out were "standard" in terms of human resources. Nonetheless, the result demonstrated the essential role of psychological support from professionals in the management of addiction. The medical domain came in third place, with 13.9% of procedures.

The intensity of management varied significantly as a function of the severity of dependence, as evaluated by the ASI. For this reason, the mean time devoted to patients could be doubled when managing severely dependent patients. However, team investment in the event of severe dependence mainly concerned psychological and social management, while medical time was not significantly modified; in addition, analysis showed that medical status itself affected neither the number of procedures nor the management time spent in this domain. It could be hypothesised that in the case of severe organic disease, patients are referred to specialised medical services, thus emphasising the limitations of the medical role of CCAA.

Chronological analysis of care helped to clarify the interaction between management and the benefits experienced by patients. The mobilisation of health professionals and patients, which was particularly intense during the first month, rapidly declined over time; benefits (in terms of the proportion of abstinent patients) reached their peak in the third month, an observation corroborated by the improvement in ASI scores during the second completion of this questionnaire. After that time, numerous patients experienced a relapse, but remobilisation of the carer-patient relationship enabled a further improvement for some of them. These data clearly demonstrate firstly, the relative transience of abstinence, and secondly, the need for teams to maintain close and regular contacts over a long period; we had previously emphasised this point during an earlier study concerning the financial costs of treatment programs for alcoholics: "as the majority of funds are spent during the initial phase of the program, outpatient care should be reinforced in order to maintain benefits and avoid money wasting" [7].

## Conclusion

Overall, this study provided a clear overview of the clinical management of a sample of individuals consulting an alcoholism outpatient centre staffed by a medical, psychological and social care team. The predominance of social and psychological components over the medical component was clearly established but it is possible that the results were biased in this setting, because of the choices made by the team carrying out the study. The severity of dependence is a factor which significantly influences the intensity of care, particularly in the psychological and social domains. Finally, the high level of recurrence observed after the third month of management, demonstrate that staffs need to maintain a constant level of care throughout the treatment process.

## Patients and methods

### Patients

Patients were recruited in two CCAA, based in two towns in the same region of south-west France (Gard). These two centres have long worked together and share some staff members. The staff in one centre includes a physician trained in addiction (0.5 full-time), a psychologist (0.5 full-time) and a social worker (full-time) who also carries out administrative tasks, while the other, larger centre is staffed by a physician (1.2 full-time), a social worker (full-time), a psychologist (0.7 full-time), a nurse (0.8 full-time) and a secretary (0.8 full-time). During the 6-month inclusion period, forty-five alcoholic patients attending one of the centres for the first time agreed to participate in the study and gave their informal consent. Because of the close links between the two centres, the data collected were combined for analysis, without account being taken of the initial site of patient recruitment.

## Methods

### Thesaurus of procedures

During a preliminary 6-month period of the study, a thesaurus of procedures was compiled by the staff members; it was then tested during a second 6-month period and validated before any patients were recruited.

Seven different sections were defined: patient interviews, family interviews, team meetings, group therapy, administrative management, support and visits. Once classified under the corresponding heading, each procedure was defined in terms of the domain of intervention (medical, psychiatric or social), the mode of communication (face to face, phone, letter, internet) and the scheduled or unscheduled nature of this procedure in the treatment programme. Once a patient had been included in the study, any procedure concerning him or her and carried out by a member of staff was coded according to the thesaurus and recorded on a standardized form. The start and completion times of procedures were also recorded, together with the patient's status with respect to alcohol (non-drinker, moderate or heavy drinker). The period of data collection was planned to last for no more than one year.

### Assessment of addiction severity

The French version of the "Addiction Severity Index" (ASI) was used for this assessment [8,9]. The index was scored by a psychologist who had received special training in its use. The ASI is a tool which includes 240 questions classified under 7 headings: medical status (M), working-income status (WI), alcohol (A), illicit drugs (D), legal status (L), familial and social environment (FS), psychological status (P). The patient's status in each domain is measured using a mathematical score based on the answers to questions in each domain: score values can range from 0 (no problem) to 1 (severe problem), and any significant reductions in score over time are assumed to constitute an improvement.

The patients were divided into two groups of moderate or severe dependence when the sum of their scores were lower than or higher than the median for the entire group studied. In the context of the study, the ASI was administered at inclusion and then three months later.

### Statistical analysis

Quantitative values were compared using the non-parametric Wilcoxon test and non-parametric test for paired series ; qualitative values were compared using the chi^2 ^test and Fisher's exact test, when necessary. The statistical significance of tests was determined by a two-tailed p-value and a level of significance of 5%. All analyses were performed using SPSS 10.0 (SPSS Inc, Chicago, Illinois, USA).

## Competing interests

The author(s) declare that they have no competing interests.

## Authors' contributions

BN conceived of the study, participated in its design and coordination and wrote the manuscript.

FM administered the ASI questionnaires

SM performed the statistical analysis performed the statistical analysis

IB, JLB and CC participated in its design and coordination

All authors read and approved the final manuscript.

**Table 2:** Intensity of care as a function of the severity of dependence

	Dependence		
	Moderate	Severe	p
No. of patients	22	23	
Follow-up (months)	8.8 ± 2.9	8.9 ± 2.6	NS
Procedures			
Sum	488	853	
m ± SD	22.2 ± 14.9	37.1 ± 24.5	0.02
Hours			
Sum	232.9	521.4	
m ± SD	10.6 ± 9.1	22.7 ± 23.6	0.005
Domain			
Psychological (mean proc.^1 ^± SD)	4.1 ± 3.7	8.7 ± 8.2	0.05
Medical	3.5 ± 1.9	4.7 ± 3.5	NS
Social	4.7 ± 4.9	11.4 ± 12.5	0.01

^1^during follow-up period.

**Table 3:** Variations in ASI scores in the 21 patients who completed ASI as second time; a negative difference corresponds to an improvement

	**Difference between final and initial ASI score**						
	**M**	**WI**	**A**	**D**	**L**	**FS**	**P**
Mean	-0,14	-0,05	-0,12	-,030	0,006	-0,10	-0,10
SD	0,29	0,19	0,19	0,05	0,10	0,27	0,21
Median	0,00	0,00	-0,12	0,00	0,00	-0,10	-0,09
Minimum	-0,72	-0,59	-0,37	-0,18	-0,24	-0,60	-0,47
Maximum	+0,34	+0,26	+0,51	+0,01	+0,27	+0,41	+0,32
							
p	0.01	NS	0.004	0.02	NS	0.08	0.04

## References

[B1] Official Journal of the French Republic, 29 December 1998, decree n°98-1229.

[B2] Gual A, Lligona A, Colom J (1999). Five-year outcome in alcohol dependence. A naturalistic study of 850 patients in Catalonia. Alcohol Alcoholism.

[B3] McLellan AT, Luborsky L, Woody GE, O'Brien CP (1980). An improved diagnostic evaluation instrument for substance abuse patients, the Addiction Severity Index. Journal of Nervous and Mental Disease.

[B4] Crouzet C, Maggia B, Nalpas B, Latorre B, Daures JP, Balmes JL (2001). Les travailleurs sociaux: des observateurs privilégiés de l'alcoolisme. Alcoologie et Addictologie.

[B5] Heide M (1995). But they are capable of change... Fifteen year experience in sociotherapeutic work with chronic alcohol-dependent women and men. Alcologia.

[B6] Peleg-Oren N, Rahav G, Teichman M (2002). Social work and the treatment of substance abuse in Israël. Journal of Social work practice in the Addictions.

[B7] Nalpas B, Combescure C, Pierre B, Ledent T, Gillet C, Playoust D, Danel T, Bozonnat MC, Martin S, Balmes JL, Daures JP (2003). Financial costs of alcoholism treatment programs: A longitudinal and comparative evaluation between four specialised centres. Alcoholism Clin Exp Res.

[B8] Martin C, Grabot D, Auriacombe M, Brisseau S, Daulouède JP, Tignol J (1996). Données descriptives issues de l'utilisation de l'Addiction Severity Index en France. Encéphale.

[B9] Daeppen JB, Burnand B, Schnyder C, Bonjour M, Pécoud A, Yersin B (1996). Validation of the Addiction Severity Index in french-speaking alcoholic patients. J Stud Alcohol.

